# Supplementary material for: Agonism of mosquito and human transient receptor potential ankyrin 1 (TRPA1) channels by the natural drimane sesquiterpene cinnamodial
Source: bioRxiv. 2025 Dec 11:2025.12.09.693172. Preprint. [Version 1] doi: 10.64898/2025.12.09.693172 (PMC12713677; doi:10.64898/2025.12.09.693172)
Supplement: 1 [file NIHPP2025.12.09.693172v1-supplement-1.pdf]

bioRxiv preprint doi: https://doi.org/10.64898/2025.12.09.693172; this version posted December 11, 2025. The copyright holder for this preprint (which was not certified by peer review) is the author/funder, who has granted bioRxiv a license to display the preprint in perpetuity. It is made available under aCC-BY 4.0 International license.

Appendix A. Supplementary data

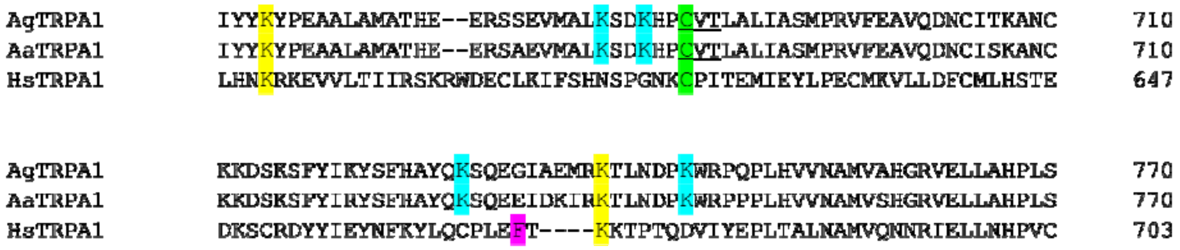

Fig. S1. Amino acid sequence alignment of the coupling domain of mosquito (Ag, Aa) and human (Hs) TRPA1 channels. Green-shaded residues correspond to a conserved Cys (683 in Ag/AaTRPA1 or 621 in HsTRPA1) predicted to be important in the covalent binding of electrophiles, including JT010 and CDIAL. Yellow-shaded residues indicate conserved Lys residues in both mosquito and human TRPA1 (656 and 738 in Ag/AaTRPA1 or 591and 671 in HsTRPA1) predicted to be important in the binding of CDIAL. Blue-shaded residues indicate Lys residues only conserved in mosquito TRPA1 (678, 681, 728, 744 in Ag/AaTRPA1) predicted to be important in binding CDIAL. Magenta-shaded residue is Phe669 that together with Cys621 contributes to binding of JT010 to HsTRPA1(Matsubara et al., 2022). Underlined residues indicate conserved 'CVT' motif of insect TRPA1 channels involved with binding of nepetalactone (Li et al., 2025). For mutated constructs of AaTRPA1 used in the present study the highlighted Cys was changed to Ser and/or all of the highlighted Lys residues were simultaneously changed to Ala.

bioRxiv preprint doi: https://doi.org/10.64898/2025.12.09.693172; this version posted December 11, 2025. The copyright holder for this preprint (which was not certified by peer review) is the author/funder, who has granted bioRxiv a license to display the preprint in perpetuity. It is made available under aCC-BY 4.0 International license.

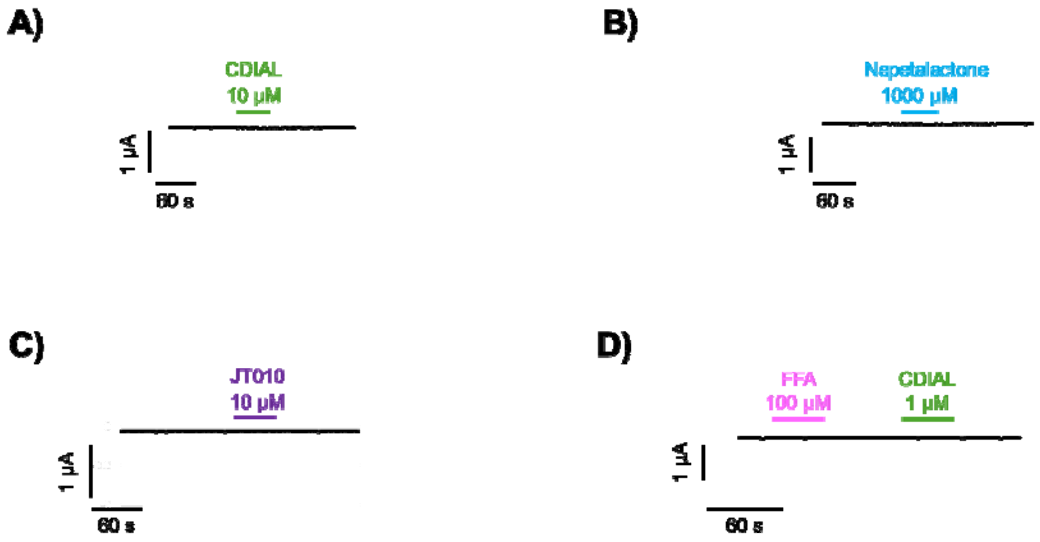

Fig. S2. Representative traces of I_m in voltage-clamped, H_2O-injected oocytes in response to CDIAL (A), nepetalactone (B), JT010 (C), or FFA and CDIAL (D). In panels A-C, only the highest concentration used in the corresponding concentration-response experiment is shown. In all cases, the ΔI_m responses were below detectable limits.

bioRxiv preprint doi: https://doi.org/10.64898/2025.12.09.693172; this version posted December 11, 2025. The copyright holder for this preprint (which was not certified by peer review) is the author/funder, who has granted bioRxiv a license to display the preprint in perpetuity. It is made available under aCC-BY 4.0 International license.

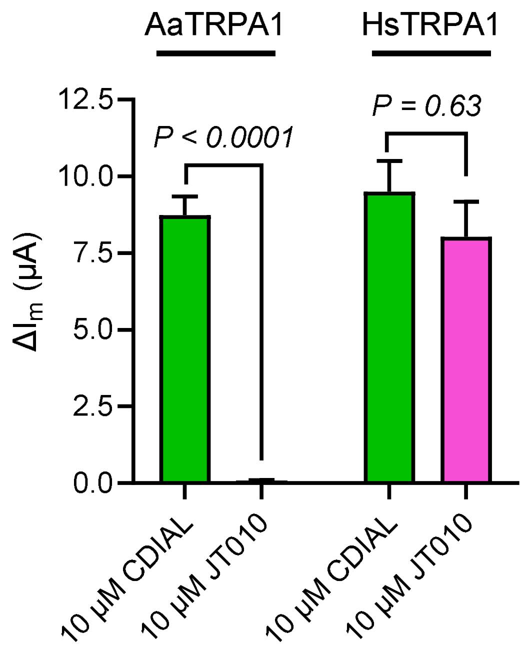

Fig. S3. Comparisons of the agonistic responses induced by 10 μM CDIAL and 10 μM JT010 in AaTRPA1 and HsTRPA1 oocytes. Values are means ± SEM, based on independent oocytes from the same batch (in AaTRPA1, n = 3 for CDIAL and n = 5 for JT010; in HsTRPA1, n = 3 for CDIAL and n = 4 for JT010). For AaTRPA1, *P* value is derived from an unpaired t-test in Prism for AaTRPA1 (data were normally distributed as determined by a Shapiro-Wilk test). For HsTRPA1, *P* value is derived from a Mann-Whitney test in Prism (data were not normally distributed as determined by a Shapiro-Wilk test).

bioRxiv preprint doi: https://doi.org/10.64898/2025.12.09.693172; this version posted December 11, 2025. The copyright holder for this preprint (which was not certified by peer review) is the author/funder, who has granted bioRxiv a license to display the preprint in perpetuity. It is made available under aCC-BY 4.0 International license.

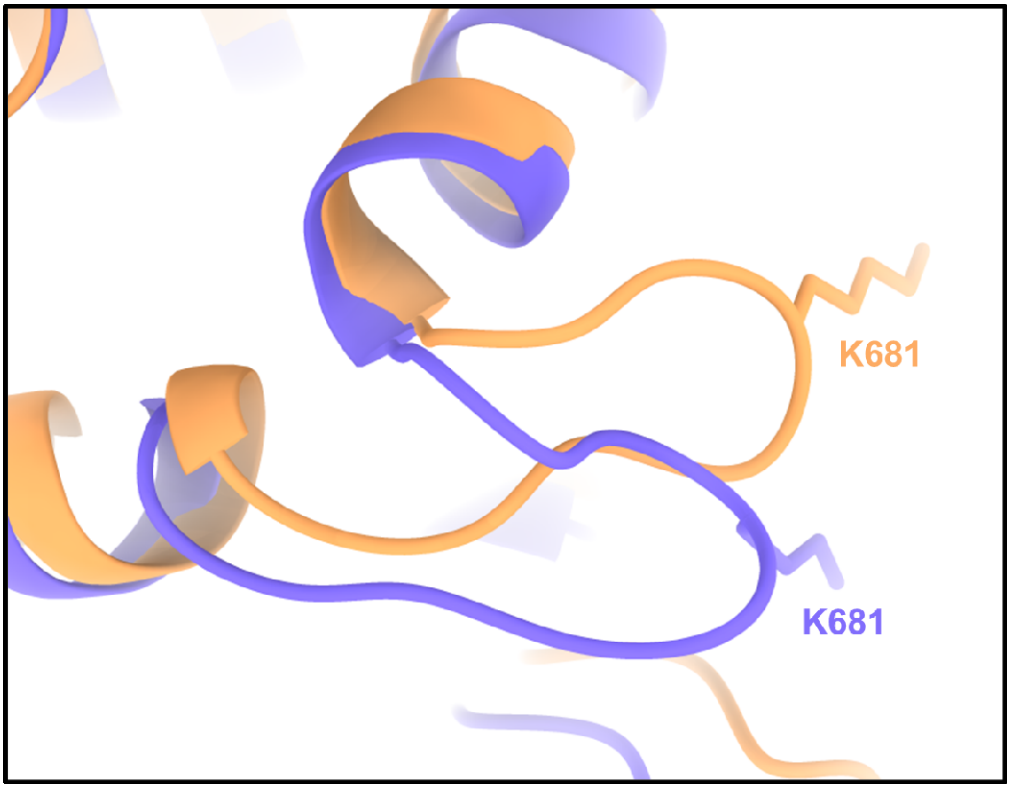

Fig. S4. Local conformational shift induced by covalent binding of CDIAL. Orange represents the AF3 predicted apo AaTRPA1 structure, while the purple represents the AaTRPA1-CDIAL covalent complex.
